# Exogenous H_2_S Ameliorates High Salt-Induced Hypertension by Alleviating Oxidative Stress and Inflammation in the Paraventricular Nucleus in *Dahl S* Rats

**DOI:** 10.1007/s12012-022-09729-7

**Published:** 2022-02-18

**Authors:** Yingying Liao, Yuanyuan Fan, Qinglin He, Yuwei Li, Dongdong Wu, Enshe Jiang

**Affiliations:** 1 Institute of Nursing and Health, Henan University, Kaifeng, China; 2 Henan International Joint Laboratory for Nuclear Protein Regulation, Henan University, Kaifeng, China; 3 School of Life Sciences, Henan University, Kaifeng, China

**Keywords:** H_2_S, PVN, Oxidative stress, Inflammation, NF-κB, Apoptosis

## Abstract

Hydrogen sulfide (H_2_S) is an important gaseous signaling molecule that regulates cardiovascular activity in animals. The hypothalamic paraventricular nucleus (PVN) is a major integrative region involved in blood pressure (BP) regulation. We explored whether exogenous H_2_S application by intraperitoneal injection of sodium hydrosulfide (NaHS) alleviates BP increase induced by a high salt diet (HSD) and the role of PVN in *Dahl* salt-sensitive (*Dahl S*) rats. *Dahl S* rats were divided into four groups according to diet regime (normal salt diet [NSD] and HSD) and treatment method (daily intraperitoneal NaHS or saline injection). We monitored BP, food and water intake, and body weight for 8 weeks. Plasma, kidney, and brain tissues were collected at the end of the experiment. We found that exogenous H_2_S not only delayed BP elevation but also attenuated the increase in the levels of norepinephrine, cystatin C, and blood urea nitrogen in the plasma of *Dahl S* rats with an HSD. Furthermore, H_2_S enhanced the total antioxidant capacity, superoxide dismutase, and glutathione peroxidase in the PVN. Exogenous H_2_S attenuated the protein expression of the nuclear factor-κB pathway and proinflammatory cytokines, which were significantly higher in the PVN in rats with an HSD than in rats with an NSD. Additionally, exogenous H_2_S relieved PVN neuronal apoptosis induced by an HSD. These findings suggest that exogenous H_2_S attenuates hypertension caused by an HSD by ameliorating oxidative stress, inflammation, and apoptosis in the PVN. This study provides evidence of the benefits of peripheral H_2_S therapy for hypertension.

## Introduction

Hypertension is a type of cardiovascular and cerebrovascular disease that is characterized by a continuous elevation of blood pressure (BP) that results in associated serious medical illness [1]. According to the report on “Nutrition and Chronic Disease Status of Chinese Residents (2020)” in China in 2019, the death rate due to chronic diseases, such as cerebrovascular and cardiovascular diseases, was as high as 80.7%. In addition, hypertension is considered the primary risk factor for stroke in China [1, 2]. Accumulating evidence indicates that nutritional lifestyles, such as a high salt diet (HSD), are one of the most important factors that contribute to the development of hypertension.

It is well established that the hypothalamic paraventricular nucleus (PVN) is an important nucleus for the central regulation of cardiovascular activity. It contains rich autonomic neurons that participate in the regulation of sympathetic nerve activity and BP [3, 4]. Neuronal activation of the PVN can increase the neuro-excitatory response, raise BP, and increase heart rate under conditions of stress [3, 4].

Over the past several years, hydrogen sulfide (H_2_S) has been recognized as a toxic and hazardous gas for the human body owing to the discovery of several features, such as olfactory paralysis, sudden loss of consciousness, pulmonary edema, mucosal irritation, and keratoconjunctivitis in people exposed to high concentrations of H_2_S [5, 6]. In the past few decades, increasing evidence has shown that H_2_S plays a positive role in the regulation of crucial physiological functions, such as hippocampal activity and muscle relaxation. H_2_S in low concentrations plays a protective role against many diseases [7]. Like carbon monoxide (CO) and nitric oxide (NO), H_2_S plays an important physiological role as a signaling molecule and provides therapeutic effects in the treatment of a wide range of diseases [8, 9]. Endogenous H_2_S is mainly present in mammals and is catalyzed by three enzymes, cystathionine β-synthase (CBS), cystathionine-γ-lyse, and 3-mercaptopyruvate sulfur transferase, of which CBS is the primary enzyme involved in neuromodulation [10]. A recent study found that microinjection of CBS inhibitors, hydroxylamine or amino-oxyacetate, into the rostral ventrolateral medulla produces an increase in renal sympathetic nerve activity, mean arterial pressure (MAP), and heart rate [11].

A study has shown that a high-fat diet (HFD) can lower the biosynthesis of H_2_S in the liver, kidneys, and lung and increase plasma interleukin (IL)-6, IL12p40, and granulocyte colony-stimulating factor (G-CSF) levels in mice [12]. In another study, Wu et al. [13] found that exogenous H_2_S application by intraperitoneal injection of sodium hydrosulfide (NaHS), a donor of H_2_S, reduced not only the degree of kidney fibrosis but also the expression level of tumor necrosis factor-α (TNF-α), IL-6, monocyte chemoattractant protein-1, and nuclear factor-kB (NF-kB) subunits p50, p65, and phosphorated p65 (p-p65) in the kidneys of HFD-induced obese mice. That study suggested that H_2_S mitigates renal injury via the reduction of kidney inflammation in HFD-induced obese mice. H_2_S also plays an important role in alleviating hypertension and kidney damage induced by an HSD in *Dahl* salt-sensitive (*Dahl S*) rats. Exogenous H_2_S application by intraperitoneal injection of NaHS inhibits myocardial hypertrophy in HSD-stimulated *Dahl S* rats by enhancing antioxidant capacity and inhibiting oxidative stress in myocardial tissues. It also protects against HSD-induced renal damage by enhancing antioxidant capacity and inhibiting renal oxidative stress [14, 15]. In contrast, HSD increases MAP and induces an inflammatory state in the PVN of *Dahl S* rats [16]. Endogenous or exogenous H_2_S changes by microinjection of hydroxylamine hydrochloride or GYY4137 into the PVN attenuates sympathetic activity and hypertensive responses partly by decreasing reactive oxygen species (ROS) and proinflammatory cytokines (PICs) within the PVN in rats with HSD [17]. However, further investigation of the effects of intraperitoneally injected NaHS on BP increase induced by HSD is needed to determine the central mechanism of PVN neuronal activity underlying BP changes.

This study aimed to explore whether exogenous H_2_S exposure via intraperitoneal NaHS injection influences BP increase induced by an HSD and determine the role of PVN in such BP changes using adult *Dahl S* rats. Findings from these experiments contribute to our understanding of the role of H_2_S treatment in peripheral tissue for hypertension associated with lifestyle-based eating habits, such as high salt intake.

## Materials and Methods

### Subjects

Eight-week-old male *Dahl S* rats were purchased from the Charles River Laboratory Animal Technology Co. Ltd. (Beijing, China) and allowed to acclimate for 1 week before the experiment. They were fed in the Animal Center in the School of Life Sciences, Henan University. All rats were maintained on a 12-h light/dark cycle and had free access to food and water before the experiment. The experimental protocols were approved by the Animal Care and Use Committee of the College of Medicine, Henan University (Ethical Clearance No. HUSOM2020-299). The design of the experiment conformed to the Guide for the Care and Use of Laboratory Animals published by the United States National Institutes of Health.

### Experimental Design

Twenty-four *Dahl S* rats were divided into four groups according to diet type (normal salt diet [NSD] or HSD) and treatment (intraperitoneal saline or NaHS injection): (1) NSD group: rats were fed with food containing 0.3% sodium chloride (NaCl) and intraperitoneally injected with saline daily; (2) NSD + NaHS group: rats were fed with food containing 0.3% NaCl and injected intraperitoneally with NaHS daily; (3) HSD group: rats were fed with food containing 8% NaCl and intraperitoneally injected with saline daily; (4) HSD + NaHS group: rats were fed with food containing 8% NaCl and intraperitoneally injected with NaHS daily. Rats in the NSD + NaHS and HSD + NaHS groups were given 90 μmol/kg/day NaHS by intraperitoneal injection daily. The amount of the NaHS application was administered according to the methods of Huang et al. [14]. The rats were treated for 8 weeks, and food and water intake and body weight (BW) were monitored weekly.

### Blood Pressure Measurements and Tissue Collection

BP and heart rate of all rats were measured while conscious using the tail-cuff method on a non-invasive BP system from Zhongshi Technology (Beijing, China) following manufacturer instructions. Each rat was acclimatized for restraint in the tail-cuff tube for 15 min/day over 3 consecutive days. After adaptation training, BP and heart rate of each rat were measured using the tail-cuff method for another 3 consecutive days. Average BP and heart rate values were used as a baseline for all rats. Subsequently, rats were given different diets and treatments. BP and heart rate were measured three times each week, and these measurements were repeated for 8 weeks. The average of the three BP and heart rate values each week were considered the BP value of each rat for that week. On the last day of the experiment, 24-h food intake, water intake, and BW were measured. Rats were then euthanized, and tissue samples of the blood, heart, kidneys, and brain were collected. The weights of the heart and kidneys of each rat were measured after collection. Trunk blood was collected in chilled tubes containing ethylenediaminetetraacetic acid (2 mg/ml) from the abdominal aorta of rats and centrifuged for 30 min at 4 °C. The plasma was transferred to a 2 ml centrifuge tube and stored in a – 80 °C freezer to measure the level of circulating norepinephrine (NE), blood urea nitrogen (BUN), and cystatin C (Cys-C) using the enzyme-linked immunosorbent assay (ELISA) kit following manufacturer guidelines. For the PVN tissue collection, the methods were referenced from Larson et al. [18]. In brief, male Dahl S rats were euthanized at the end of the experiment. The brains were removed quickly, and the hypothalamic PVN was punched out using a 1-gauge needle (1.5 mm inner diameter). To identify the PVN tissue, the optic tract was identified, and an approximately 1-mm-thick brain section was taken from the rostral endpoint of the optic tract. Samples were frozen in liquid nitrogen and stored at − 80 °C until used for further molecular analysis.

### Hematoxylin–Eosin and Masson Staining of the Renal Structure

The kidney was fixed in 4% paraformaldehyde solution and then underwent paraffin embedding. An 8-μm tissue specimen was taken from a transverse kidney section for hematoxylin–eosin (HE) and Masson staining in strict accordance with the experimental steps. A Leica image processing and analysis system was used for image acquisition. The images were qualitatively analyzed to determine structural changes in the kidneys.

### Immunohistochemical and Immunofluorescent Studies of PVN

The rats were anesthetized with sodium pentobarbital and transcardially perfused with saline, followed by 4% paraformaldehyde solution. The brains were removed and immersed immediately in 4% paraformaldehyde for 24 h and subsequently transferred to 30% sucrose solution until the brain was deposited in the bottom of the bottle. We collected 20-μm coronal sections containing the PVN. ROS in the PVN were detected using fluorescent-labeled dihydroethidium staining and visualized using a confocal laser-scanning microscope (Leica, Wetzlar, Germany). Fluorescence intensity for DHE in the PVN was analyzed and quantified using the Image J software (NIH, Bethesda, USA). For the immunofluorescence staining of PICs, including TNF-α, IL-6, and IL-1β, in the PVN, brain coronal sections (20 μm) containing the PVN were first washed in phosphate-buffered saline (PBS) three times for 10 min each. Sections were incubated with 5% horse serum in PBS for 1 h, and then incubated with mouse anti-TNF-α antibodies (1:100 dilution, Santa Cruz Biotechnology, Dallas TX, USA), mouse anti-IL-6 antibodies (1:100 dilution, Abcam), or mouse anti-IL-1β antibodies (1:100 dilution, Santa Cruz Biotechnology) in PBS containing 0.5% Triton X-100 and 5% horse serum for 72 h at 4 °C. Subsequently, sections were washed with PBS three times for 10 min each, followed by incubation with a secondary antibody: Alexa Fluor® 488 donkey anti-mouse immunoglobulin G (IgG; 1:1000) or Alexa Fluor® 594 donkey anti-mouse IgG (1:1000) overnight at 4 °C. The sections were mounted on microslides, and images were acquired using a Leica microscope and quantified using the Image J software. The method of quantification of immunoreactivity of PICs within the PVN is described in a previous study [16].

### The ELISA Measurement of Glutathione Peroxidase, Total Antioxidant Capacities, Superoxide Dismutase, and Malondialdehyde in the PVN

The activity of glutathione peroxidase (GSH-Px), total antioxidant capacities (T-AOC), superoxide dismutase (SOD), and malondialdehyde (MDA) in the PVN was measured using commercial ELISA kits (Nanjing Jiancheng, Nanjing China) according to manufacturer instructions.

### Western-Blotting Measurement of the NF-κB Pathway- and Apoptosis-Associated Proteins in the PVN

The PVN tissues were collected quickly, submerged in liquid nitrogen, and stored at − 80 °C. The total protein was extracted from the PVN tissues, and western-blotting was performed to detect the target proteins. The volume of protein was loaded according to the results from the preliminary experiment of protein detection following the western-blotting procedure after protein quantification with bicinchoninic acid (BCA) protein assay kit. The sample loading volume of the protein involved was 80 µg in the NF-kB pathway and 60 µg in the apoptosis pathway. The primary antibodies, including anti-p50, anti-p65, anti-p-p65, anti-Bax, anti-Bcl-2, anti-cleaved-caspase3, and anti-cleaved poly ADP-ribose polymerase (PARP) were incubated at 4 °C overnight. Secondary antibodies were incubated at room temperature for 1 h to detect the proteins involved in NF-kB and apoptosis signaling. The final gel concentration chosen in this study was 12% according to the protein molecular weight and the band separation results. The species in which each primary antibody was raised was the rabbit, and the species in which the primary antibody was raised for incubating the reference protein β-actin was the mouse. The results were normalized to the level of β-actin. The reaction was detected with an enhanced chemiluminescence system (Thermo Scientific, MA, USA), and the band was semi-quantified using the Image J software.

### Transferase-Mediated dUTP Nick End Labeling Staining of the Neurons in the PVN

Terminal deoxynucleotidyl transferase-mediated dUTP nick end labeling (TUNEL) was applied to evaluate neuronal apoptosis using a cell death detection kit (Servicebio, Wuhan, China) following manufacturer instructions. Briefly, 8-μm brain slices containing PVN were fixed on slides for 30 min with 4% paraformaldehyde. They were then permeabilized with 0.1% Triton X-100 and incubated with a 50-μl TUNEL reaction mixture for 60 min at 37 °C in darkness. The slices were then rinsed three times with PBS. After counterstaining with 5 mg/ml 4′,6-diamidino-2-phenylindole for 5 min at room temperature, the brain slices were photographed using a fluorescent microscope (Leica DMIL, German). The percentages of TUNEL-positive cells were calculated using the formula: apoptotic index = (positively stained apoptotic cells)/(total number of cells) × 100%.

### Reagents and Antibodies

The reagents and antibodies used in the current study are outlined in Table 1. Anti-TNF-α and anti-IL-1β antibodies were purchased from Santa Cruz Biotechnology (Dallas, USA). Anti-IL-6 and anti-β-actin antibodies and Alexa Fluor® 488 donkey anti-mouse IgG were purchased from Abcam (Cambridge, UK). Alexa Fluor® 594 donkey anti-mouse IgG were purchased from Jackson ImmunoResearch Labs (West Grove, USA). Anti-p65, p-p65 (Ser536), anti-p50, anti-Bax, anti-Bcl-2, anti-cleaved-caspase3, and anti-cleaved-PARP antibodies were purchased from Absin (Shanghai, China). Goat anti-rabbit IgG (H&L) secondary antibodies were purchased from Bio-Techne China (Shanghai, China). ROS, total antioxidant capacity, SOD, GSH-Px, and MDA assay kits were purchased from Nanjing Jiancheng Bioengineering Institute (Nanjing, China). NaHS hydrate was purchased from Sigma-Aldrich LLC (St. Louis, MO, USA). The TUNEL cell apoptosis detection kit was purchased from Wuhan Servicebio Technology (Wuhan, China). The rat noradrenaline (NA) ELISA Kit and Cys-C ELISA kit were purchased from CUSABIO Technology LLC (Houston, USA). The BUN test kit was purchased from Rayto (Shenzhen, China).

**Table 1:** Information of the products used in the study

Reagent	Company	Catalog #	Source	Dilutions for WB	Dilutions for IF
Primary antibodies					
Anti-TNF-α	Santa Cruz	Sc-52746	Mouse	/	1:100
Anti-IL-6	Abcam	Ab9324	Mouse	/	1:100
Anti IL-1β	Santa Cruz	Sc-52012	Mouse	/	1:100
Anti-p65	Absin	Abs131170	Rabbit	1:500	/
Phospho-p65	Absin	Abs130624	Rabbit	1:500	/
Anti-p50	Absin	Abs146717	Rabbit	1:500	/
Anti β-actin	Abcam	Ab8224	Mouse	1:10,000	/
Anti-Bax	Absin	Abs130057	Rabbit	1:500	/
Anti-Bcl-2	Absin	Abs131701	Rabbit	1:500	/
Anti-cleaved-caspase3	Absin	Abs132005	Rabbit	1:500	/
Anti-cleaved-PARP	Absin	Abs132006	Rabbit	1:500	
Secondary antibodies					
Anti-mouse 488	Abcam	Ab150159	Donkey	1:1000	/
Anti-mouse 594	Jackson	NC0322938	Donkey	1:1000	/
Anti-rabbit IgG (H&L)	Novusbio	NB7160	Goat	1:5000	/
Other kits					
BCA (bicinchoninic acid) protein assay kit	Epizyme	ZJ101	/	/	/
Reactive oxygen species (ROS) assay kit	Nanjing Jiancheng	E004-1-1	/	/	/
Total antioxidant capacity assay kit	Nanjing Jiancheng	A015-1-2	/	/	/
Superoxide dismutase (SOD) assay kit	Nanjing Jiancheng	A001-3-2	/	/	/
Glutathione peroxidase (GSH-PX) assay kit	Nanjing Jiancheng	A005-1-2	/	/	/
Malondialdehyde (MDA) assay kit	Nanjing Jiancheng	A003-1-2	/	/	/
Sodium hydrosulfide hydrate (NaHS)	Sigma	207683-19-0	/	/	/
Tunnel cell apoptosis detection kit	Servicebio	G1501	/	/	/
Rat noradrenaline (NA) ELISA kit	CUSABIO	CSB-E07022r	/	/	/
Blood urea nitrogen (BUN) test kit	Rayto	S03036	/	/	/
Rat cystatin C (Cys-C) ELISA kit	CUSABIO	CSB-E08385r	/	/	/

*WB* western blotting, *IF* immunofluorescence

### Statistical Analysis

All data are presented as means ± standard errors of the mean. For the BP data analysis, a two-way repeated-measures analysis of variance (ANOVA) was used, followed by Tukey’s post hoc tests. For other data analyses, differences between groups were determined using two-tailed Student’s *t*-tests using the GraphPad Prism 8.0 software (San Diego, CA, USA). A *P* < 0.05 was considered statistically significant.

## Results

### Exogenous H_2_S Delayed BP Increase Induced by an HSD in *Dahl S* Rats

**Fig. 1:**
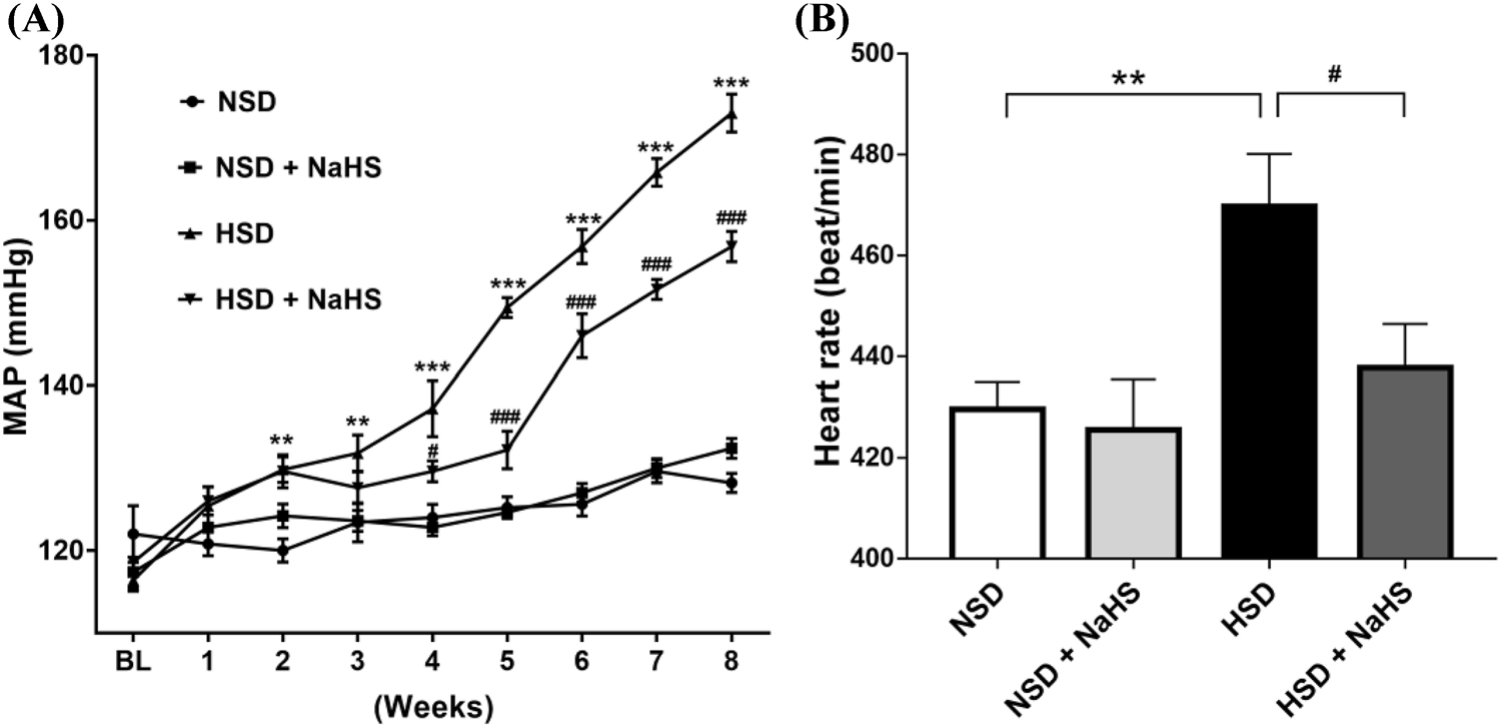
H_2_S slowed down the blood pressure elevation and reduced the heart rate increase induced by high salt diet in *Dahl S* rats. **A** The change in mean arterial pressure (MAP) of rats in 4 groups during the 8-week experiment. **B** The change of heart rate in 4 groups of rats in the 8th week. Values are expressed as means ± SEM (*n* = 5). **P < .01, ***P < .001 compared with group NSD; ^#^*P* < .05, ^###^*P* < .001 compared with group HSD. *NSD* normal salt diet, *HSD* high salt diet, *NaHS* sodium hydrosulfide, *BL* baseline

We first analyzed the BP data of the four groups of rats by performing a two-way repeated-measures ANOVA. Results showed a significant main effect of group (*F*_3,16_ = 121.7, *P* < 0.001) and time (*F*_8,128_ = 122.4, *P* < 0.001), and a significant group × time interaction (*F*_24,128_ = 20.4, *P* < 0.001). Post hoc analysis was conducted for BP across the four groups for each week. Results showed that an HSD significantly increased BP in *Dahl S* rats from the second week and resulted in hypertension from week 5 in rats with HSD but not in rats with NSD. The intraperitoneal injection of NaHS reduced the BP increase induced by the HSD from the 4th week significantly more in HSD + NaHS rats than in rats in the HSD group. However, intraperitoneal NaHS injection did not affect the BP of rats in the NSD + NaHS group compared with rats in the NSD groups (NSD + NaHS vs. NSD, all *p* > 0.05 for all weeks; Fig. 1A). As shown in Fig. 1A, the BP elevation in the HSD + NaHS group slowed from week 3, and a significant difference in BP was observed in comparison with that of the HSD group from the week 4. In addition, heart rate was significantly faster in the HSD group rats than that in rats in the NSD group on the week 8 of testing. Intraperitoneal NaHS injection significantly reduced heart rate in the HSD + NaHS rats **(**Fig. 1B). Exogenous H_2_S application delayed HSD-induced BP elevation and increased heart rate, although rats could still develop hypertension over an extended period.

### Exogenous H_2_S Alleviated Kidney Damage Induced by HSD in *Dahl S* Rats

A previous study showed that an HSD could induce renal oxidative stress and kidney damage in *Dahl S* rats [15]. In this experiment, we measured relative physiological indicators, such as heart weight, kidney weight, BW, water intake, and food intake on completion of the experiment on week 8 (Table 1). We found that an HSD significantly increased heart weight, kidney weight, heart weight/BW, kidney weight/BW, and water intake in rats in the HSD group compared with those in rats in the NSD group. Intraperitoneal H_2_S application attenuated the increase in these indicators in HSD + NaHS rats significantly more than that in rats in the HSD group. No difference was observed for heart weight, kidney weight, BW, heart weight/BW, kidney weight/BW, water intake, or food intake between rats in the NSD + NaHS and NSD groups, which indicated that intraperitoneal H_2_S application did not affect the above indicators in rats with an NSD. In addition, there were no differences in BW or food intake among the four groups. This suggested that H_2_S application did not affect the growth of rats with different diets during the 8 weeks (Table 2).

**Table 2:** Changes in the relative physiological indicators at the end of week 8 of the experiment

Group	NSD	NSD + NaHS	HSD	HSD + NaHS
Heart weight (g)	1.31 ± 0.04	1.38 ± 0.11	1.64 ± 0.05***	1.49 ± 0.04^##^
Kidney weight (g)	2.95 ± 0.21	3.26 ± 0.21	3.83 ± 0.23***	3.48 ± 0.11^#^
Body weight (BW) (g)	331.60 ± 2.87	328.40 ± 26.06	325.04 ± 14.02	321.20 ± 11.99
Heart weight/BW (%)	0.38 ± 0.02	0.36 ± 0.01	0.55 ± 0.04**	0.47 ± 0.02^#^
Kidney weight/BW (%)	0.87 ± 0.07	0.88 ± 0.07	1.15 ± 0.03***	1.08 ± 0.01^##^
Water intake (g)	39.44 ± 4.43	34.18 ± 2.97	129.96 ± 11.36***	111.44 ± 7.36^#^
Food intake (g)	22.80 ± 2.32	25.06 ± 0.65	24.16 ± 1.07	23.54 ± 0.74

*BW* body weight, *NSD* normal salt diet, *HSD* high salt diet, *NaHS* sodium hydrosulfide hydrate

**P < .01, ***P < .001 compared with the NSD group

^#^*P* < .05, ^##^*P* < .01 compared with the HSD group

**Fig. 2:**
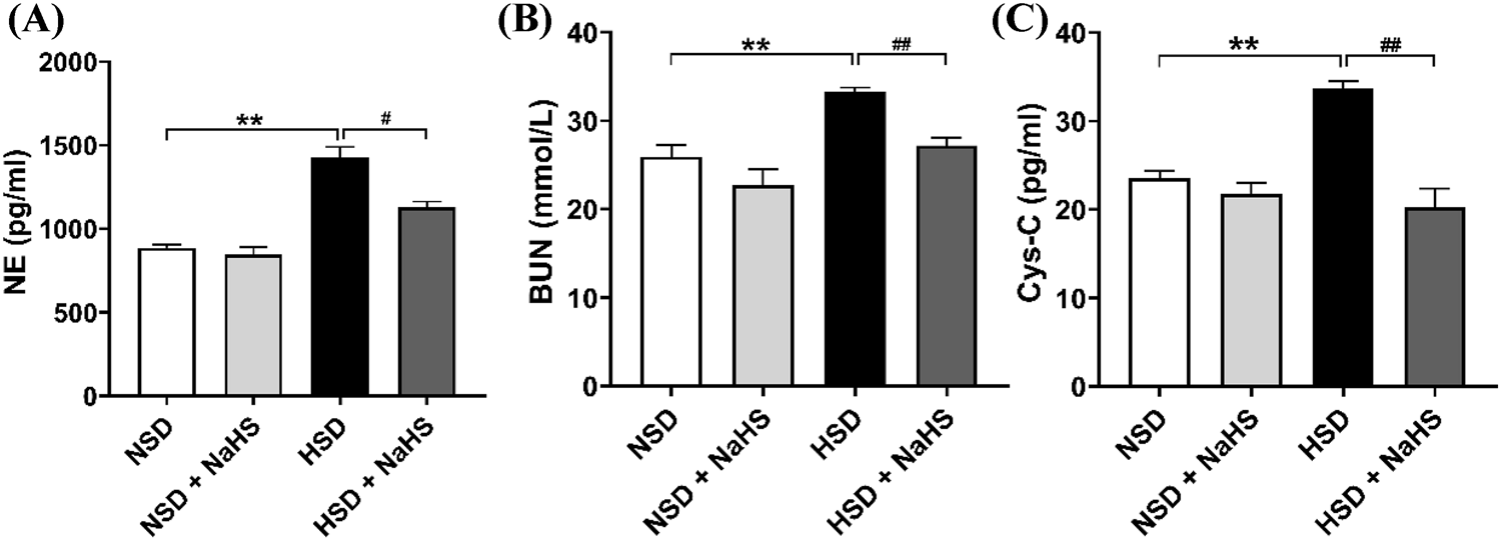
Effects of NaHS intraperitoneal injection on the level of plasma NE, BUN and Cys-C at the end of the experiment in *Dahl S* rats. **A** The changes in plasma NE level (an indirect indicator of sympathetic activity) in 4 groups of rats. **B** The changes in plasma BUN level (an indicator of kidney injury) in 4 groups of rats. **C** The changes in plasma Cys-C level (an indicator of kidney injury) in 4 groups of rats. Values are expressed as means ± SEM (*n* = 3). **P < .01, ^#^*P* < .05, ^##^*P* < .01. *NE* norepinephrine, *BUN* blood urea nitrogen, *Cys-C* cystatin C

**Fig. 3:**
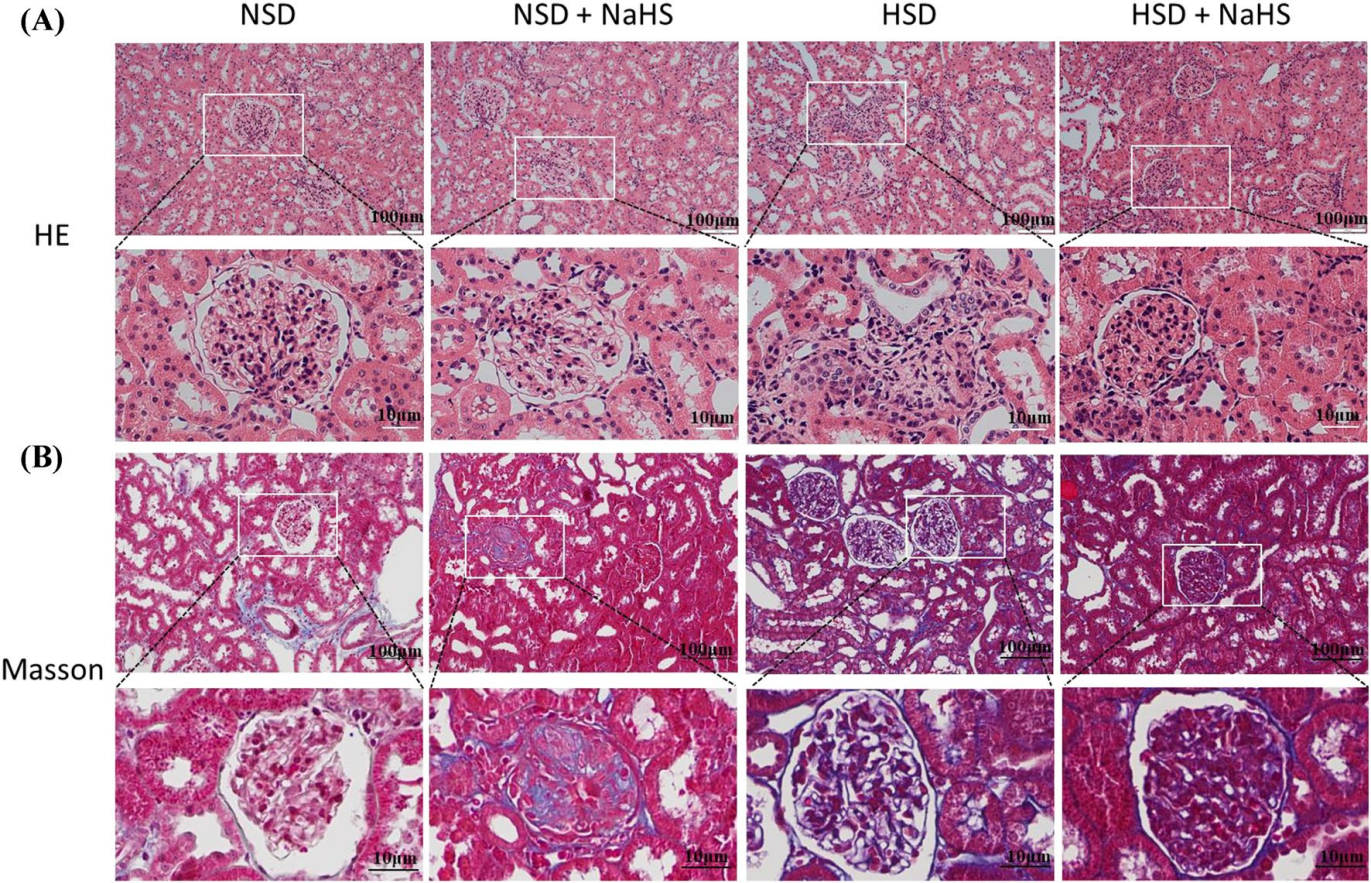
Effects of NaHS intraperitoneal injection on the glomerular structure of the kidney. **A** HE staining showed the structure of the kidney in the 4 groups of rats. The boxed rectangle in the upper panel is enlarged in the lower panel. **B** Masson staining showed the renal fibrosis in the 4 groups of rats. The boxed rectangle in the upper panel was enlarged in the lower panel. *HE* hematoxylin–eosin

The concentration of plasma NE is an important indicator that indirectly reflects the activity of the sympathetic nerves. The volume of BUN and Cys-C in the plasma are additional indicators for evaluating renal function. We tested the concentration of plasma BUN and Cys-C at the end of the experiment in all four groups. The levels of plasma NE, BUN, and Cys-C were significantly higher in HSD rats than in the NSD group, and these increases induced by the HSD were significantly inhibited with treatment with NaHS. In addition, there were no differences in the levels of plasma NE, BUN, and Cys-C between the NSD and NSD + NaHS groups (Fig. 2). Given that the increased excitation of the renal sympathetic nerve, as indicated by the higher plasma NE concentration, may damage renal function, the increase in plasma NE, BUN, and Cys-C in HSD rats suggested that HSD caused kidney damage. Exogenous H_2_S application alleviated the kidney damage induced by the HSD. These findings were supported by the evaluation of renal structures using the HE and Masson staining method. HE staining showed that the glomerular structure was more disorganized, and the borders of the glomeruli were more obscure in the rats in the HSD group compared with those of NSD rats. This renal structure disorder was reversed in rats in the HS + NaHS group, and the borders of the glomeruli became visible again. Masson staining also showed greater relief from renal fibrosis in the rats in the HSD + NaHS group compared with that in the rats in the HSD group (Fig. 3).

### H_2_S Reduced Oxidative Stress and Enhanced Anti-oxidative Capacity in the PVN in *Dahl S* Rats

**Fig. 4:**
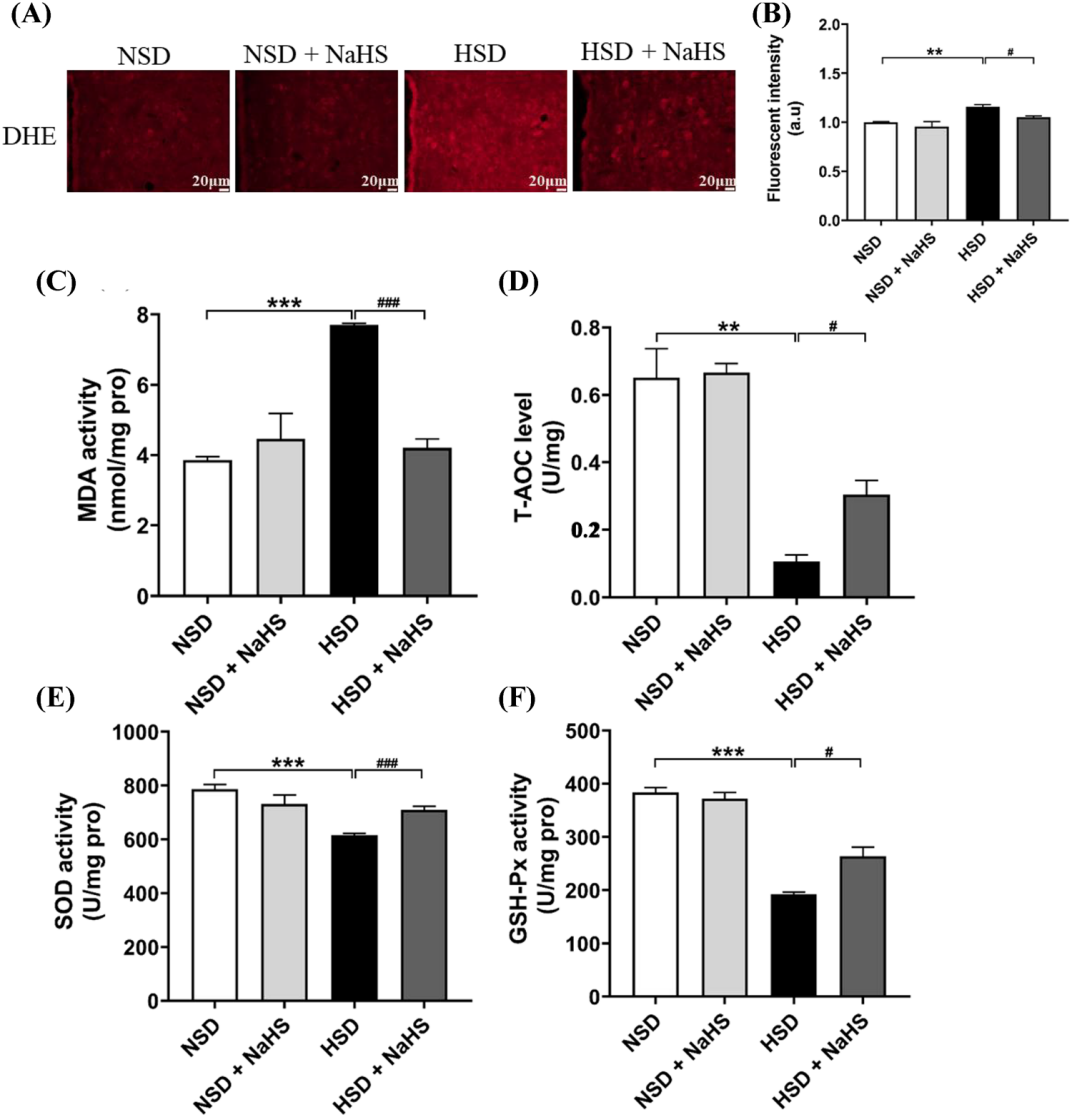
Effects of NaHS intraperitoneal injection on the oxidative stress in PVN tissue of 4 groups of rats. **A** A representative immunofluorescence image of fluorescent-labeled dihydroethidium (DHE). The images were taken under 100 × magnification. **B** The summary data of the change in DHE fluorescent intensity in PVN in 4 groups of rats. **C** The change in MDA (an indicator of peroxidase) in the PVN in 4 groups of rats. **D** The change in T-AOC activity in PVN. **E** The change in SOD activity in PVN. **F** The changes in GSH-Px activity in PVN. Values are expressed as means ± SEM (*n* = 3). **P < .01, ***P < .001; ^#^*P* < .05, ^###^*P* < .001. *ROS* reactive oxygen species, *MDA* malondialdehyde, *SOD* superoxide dismutase, *GSH-Px* glutathione peroxidase, *T-AOC* total antioxidant capacity

Changes in ROS in PVN tissues were first detected by DHE immunofluorescence staining (Fig. 4A). Results showed that exogenous H_2_S significantly reduced the increase in ROS in the PVN induced by an HSD in *Dahl S* rats (Fig. 4B). MDA is used as an indicator of lipid peroxidation of membranes. The MDA level in the PVN tissue was increased significantly more in HSD rats than in rats in the NSD group. NaHS treatment inhibited the MDA increase caused by the HSD in the rats in the HSD + NaHS group (Fig. 4C). SOD and GSH-Px are also key anti-oxidases. The levels of enzyme-driven and non-enzymatic antioxidants, such as T-AOC, SOD, and GSH-Px, in the PVN were decreased in rats in rats with HSD, but not in rats with NSD. NaHS treatment improved these antioxidants in PVN tissue in HSD + NaHS rats (Fig. 4D–F). These findings suggest that H_2_S enhances the antioxidant capacity of the PVN tissue of *Dahl S* rats with a long-term HSD.

### H_2_S Relieved the Inflammatory Response Induced by an HSD in the PVN of *Dahl S* Rats

**Fig. 5:**
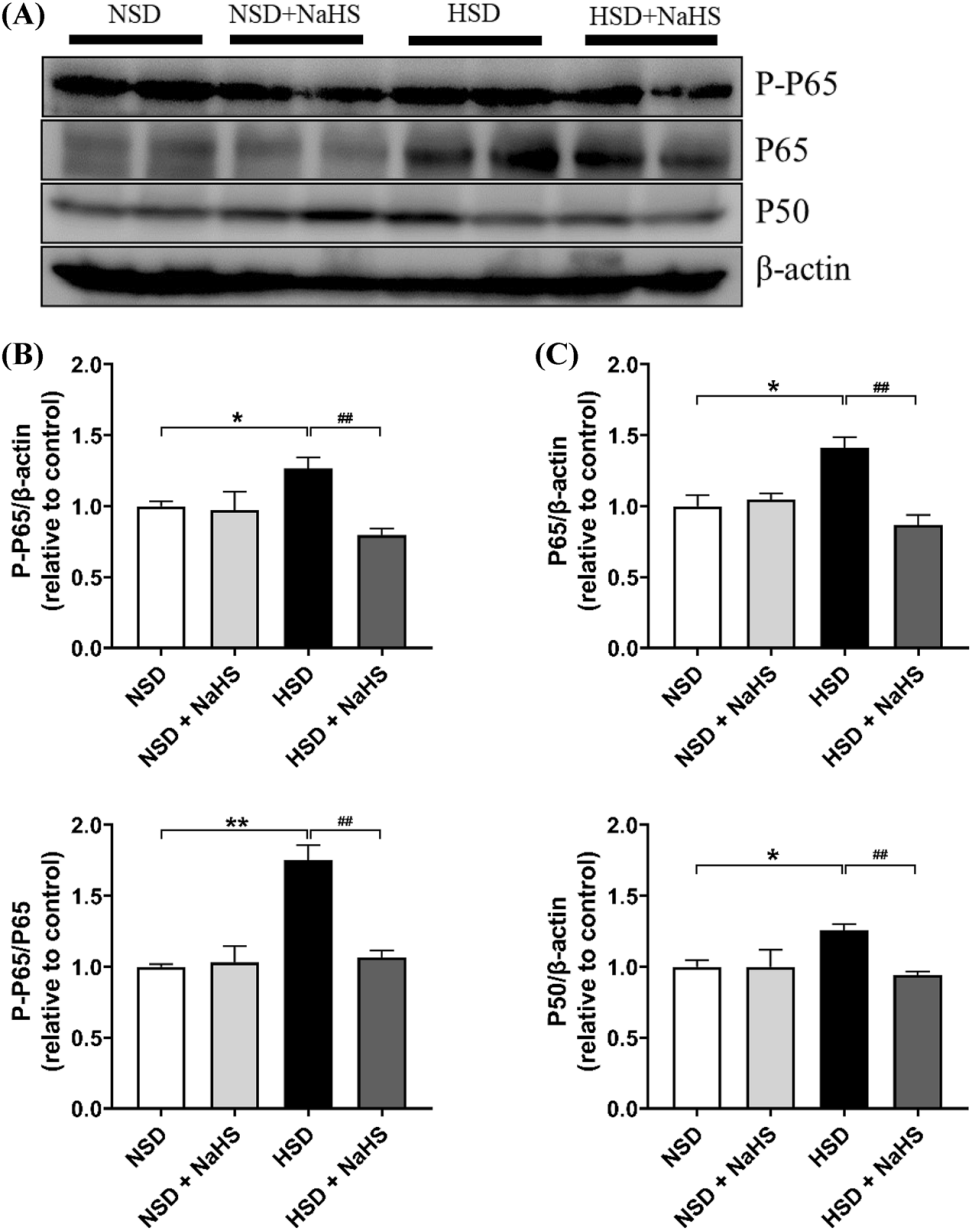
Effects of NaHS intraperitoneal injection on NF-κB signaling in PVN of *Dahl S* rats. **A** The representative immunoblots of P-P65, P65 and P50 in the PVN. It shows that NaHS intraperitoneal injection reduced the increase in the protein expression of P-P65 (**B**), P65 (**C**), P-P65/P65 (**D**) and P50 (**E**) induced by HSD in the PVN of the *Dahl S* rats. Values are expressed as means ± SEM (*n* = 3). *P < .05, **P < .01; ^##^*P* < .05

A previous study showed that an HSD induces an inflammatory response in the PVN of *Dahl S* rats [16]. In this study, we first tested whether exogenous H_2_S application ameliorates the inflammatory response induced by the HSD in *Dahl S* rats. The NF-κB signal pathway in the PVN plays an important role in developing HSD-induced hypertension [19]. Thus, we evaluated the expression level of NF-κB pathway-related proteins in the PVN using western-blotting analysis. Results showed that the expressions of p50, p65, and p-p65 were significantly upregulated in the HSD group. Exogenous H_2_S treatment significantly attenuated the increase in protein expressions of p50, p65, and p-p65 induced by the HSD in the PVN (Fig. 5). These findings suggest that the effect of H_2_S on attenuation of hypertension is due to the inhibition of the activity of the NF-κB signaling pathway in the PVN.

**Fig. 6:**
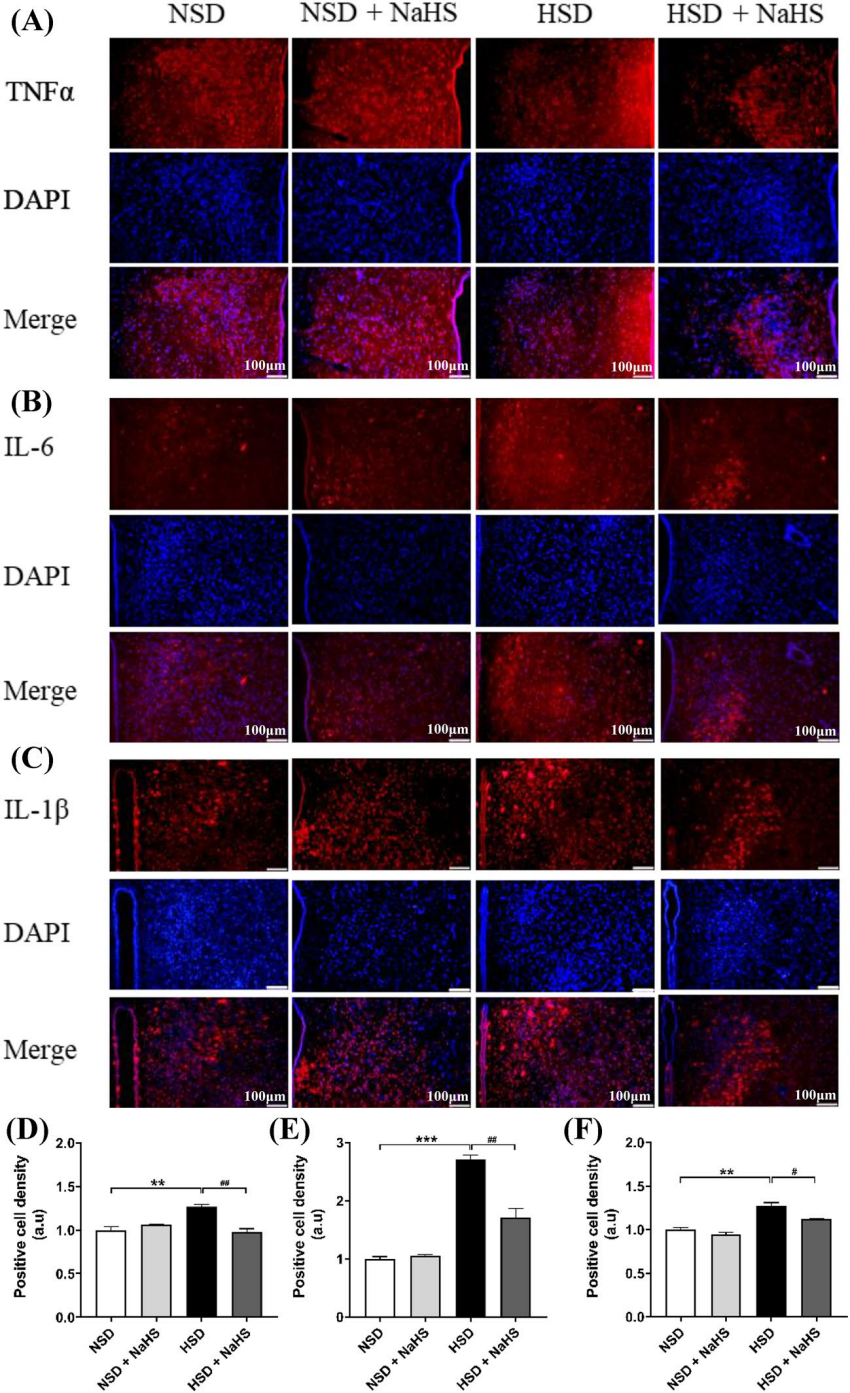
NaHS intraperitoneal injection reduced the increase in the expression of proinflammatory cytokines induced by the HSD in PVN of *Dahl S* rats. The representative immunofluorescence staining showed the expression of TNFα (**A**), IL-6 (**B**) and IL-1β (**C**) in PVN. The images were taken under 100 × magnification. Values are expressed as means ± SEM (*n* = 3). **P < .01, ***P < .001; ^#^*P* < .05, ^##^*P* < .01

Because the activated NF-κB protein may enter the cell nucleus to promote the production and expression of PICs, we assessed the expressions of TNF-α, IL-6, and L-1β in PVN tissues in the four groups of rats using immunofluorescence staining. We found that exogenous H_2_S attenuated the increased expressions of TNF-α, IL-6, and L-1β induced by the HSD in PVN tissue of *Dahl S* rats (Fig. 6). This suggested that H_2_S reduces the inflammatory response in the PVN caused by a long-term HSD in *Dahl S* rats and that exogenous H_2_S plays a protective role in the PVN inflammatory response.

### Exogenous H_2_S Alleviated Cell Apoptosis Induced by an HSD in *Dahl S* Rats

**Fig. 7:**
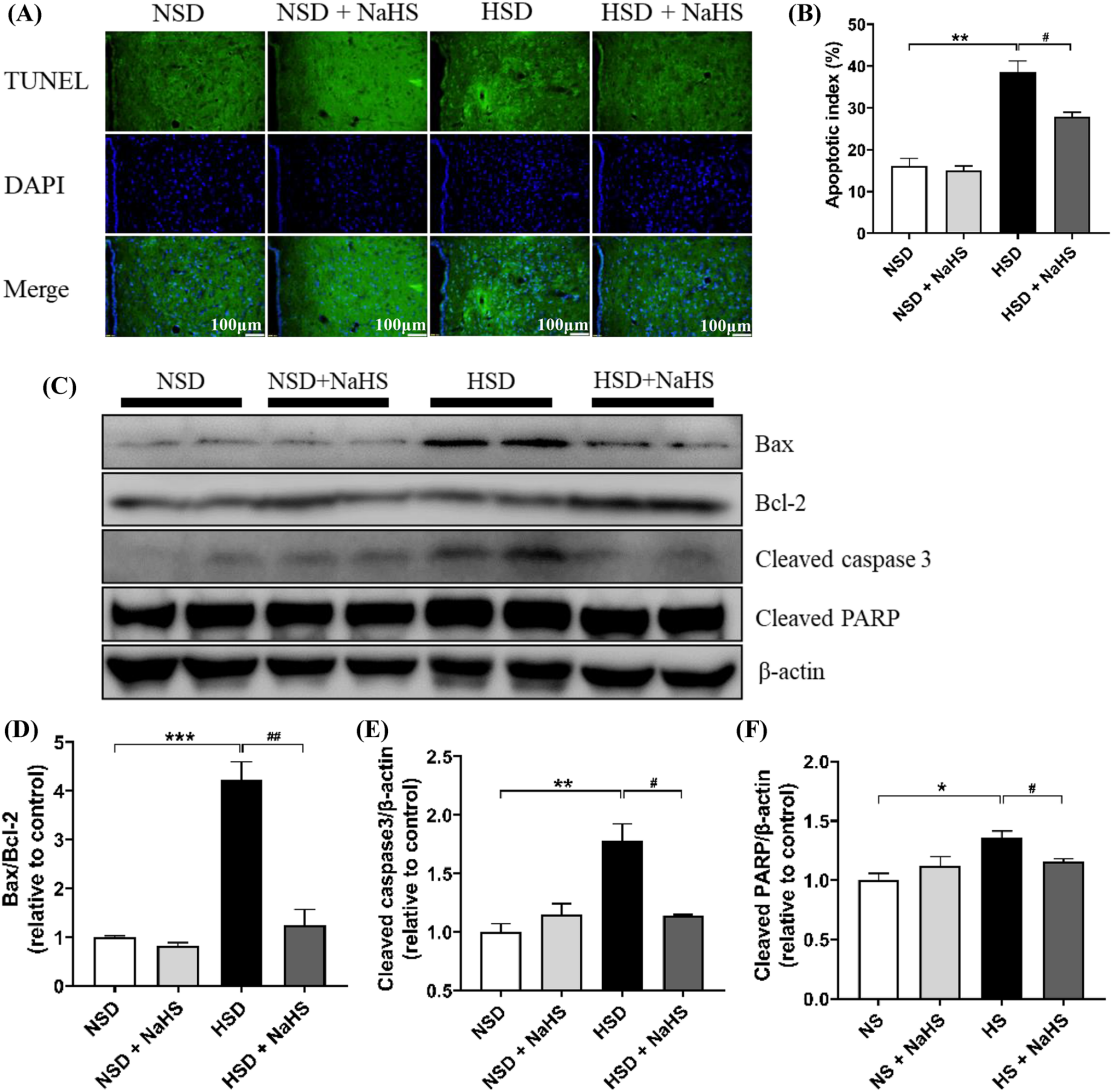
Effects of NaHS intraperitoneal injection on the cell apoptosis induced by HSD in PVN of *Dahl S* rats. **A** The apoptotic levels were measured by TUNEL staining; original magnification, × 100. **B** The summary data for the apoptotic index in PVN of the 4 groups of rats. The percentages of TUNEL-positive cells were calculated by the following formula: the apoptotic index = (positively stained apoptotic cells)/(total number of cells) × 100%. **C** The representative immunoblots of Bax, Bcl-2, Cleaved caspase3, and Cleaved-PARP in the PVN. It shows that NaHS intraperitoneal injection attenuated the increase in the protein expression of Bax/Bcl-2 (**D**), cleaved-caspase3 (**E**) and cleaved PARP (**F**) in the PVN of the *Dahl S* rats. Values are expressed as means ± SEM (*n* = 3). *P < .05, **P < .01, ***P < .01; ^#^*P* < .05, ^##^*P* < .01

We evaluated the effect of exogenous H_2_S on cell apoptosis in the PVN. An HSD significantly increased the apoptotic index and the expression of Bax/Bcl-2, cleaved-caspase3, and cleaved-PARP in the PVN of rats, which suggested that an HSD induces cell apoptosis in the PVN. However, exogenous H_2_S significantly attenuated the expression levels of these apoptotic proteins (Fig. 7), which suggests that exogenous H_2_S has a protective effect on neurons in the PVN.

## Discussion

The present study produced three key findings: (i) exogenous H_2_S application by intraperitoneal injection delayed the HSD-induced BP increase in *Dahl S* rats by alleviating peripheral tissue damage in the heart and kidneys; (ii) exogenous H_2_S application attenuated oxidative stress, protein expression of the NF-κB pathway, and production of PICs in the PVN of *Dahl S* rats; and (iii) exogenous H_2_S application reduced cell apoptosis of the PVN induced by an HSD in *Dahl S* rats. During the development of hypertension induced by an HSD in *Dahl S* rats, the kidneys and PVN are the two main target organs that are significantly impacted. Similar to other bio-gaseous molecules, such as CO and NO, H_2_S has been considered as a novel molecule that can be used to treat high BP and kidney damage caused by an HFD [13, 20]. Exogenous H_2_S application plays a protective role in the development of hypertension by reducing oxidative stress and attenuating the inflammatory response and cell apoptosis, which have an impact on the kidneys and PVN function.

Previous study have shown that exogenous H_2_S application decreases elevated BP by inhibiting HSD-induced excessive oxidative stress and kidney damage in *Dahl S* rats [15]. However, it is not known whether this method of H_2_S application delays the increase in BP and the development of hypertension via a central mechanism by affecting PVN neuronal activity. In this study, we focused on the PVN because it is a key brain nucleus in the hypothalamus for regulating sympathetic outflow, heart rate, BP, food intake, and water intake [3, 16, 21]. PVN neurons integrate signals from circumventricular organs and other cardiovascular-relevant brain regions, convey information to the rostral ventrolateral medulla or directly to the spinal cord, control the sympathetic activities, and finally regulate the function of the heart and kidneys. It should be noted that PVN neuronal activity can also be modulated by multiple factors, such as angiotensin II, glutamate, PICs, and other gas signal molecules, including NO, CO, and H_2_S. Numerous studies have shown that PVN plays an important role in the development of salt-sensitive hypertension. Liang et al. investigated the effects of direct microinjection of GYY4137, a donor of H_2_S, into the PVN on BP changes in *Dahl S* rats on an HSD. They found that the application of H_2_S into the PVN attenuated BP elevation by regulating oxidative stress and the inflammatory response [17]. We have further answered the above question, whereby exogenous intraperitoneal H_2_S application delays hypertension development by protecting peripheral organs, such as the kidneys, and the central brain tissue of the PVN, and by reducing oxidative stress, expression of the NF-κB pathway, PIC activity, and neuronal apoptosis.

This study confirmed findings of the protective role of H_2_S for heart and kidney damage in *Dahl S* rats and other animals. We found that exogenous H_2_S application reduced the increase in heart weight, heart weight/BW, and heart rate caused by an HSD. This findings are consistent with findings that exogenous H_2_S application inhibits myocardial hypertrophy in HSD-induced *Dahl S* rats [15]. The kidneys were also damaged in rats with HSD, with increased renal fibrosis as reported in this and other studies [22, 23]. An HSD-induced glomeruli structure disorder and renal fibrosis and caused disordered renal function as evidenced by the increase in plasma NE, BUN, and Cys-C. The level of NE in the plasma is an indirect indicator of sympathetic nerve activity, and previous findings have shown that an HSD increases sympathetic nervous activity, as measured by circulating NE [17, 24]. H_2_S treatment reversed the increase in these indicators. Therefore, these findings strengthen previous reports of the protective role of H_2_S against HSD-induced renal damage [14, 25–28].

To date, the mechanisms underlying the protective role of H_2_S against HSD-induced hypertension have remained unclear. H_2_S has a powerful antioxidant effect, mainly owing to its ability to directly remove ROS and downregulate enzymes that produce ROS [29]. It is primarily used as an antioxidant and anti-neuritis drug and plays a protective role in the nervous system by preventing neuronal damage caused by hypoxia [30–32]. In the brain, the PVN is a key area that controls sympathetic outflow, BP, and salt-sensing mechanisms. H_2_S in the PVN delays the occurrence of hypertension in response to an HSD and protects cell growth under conditions of oxidative stress [17]. The main characteristics of oxidative stress are an increase in the production of oxidants and impaired antioxidant defense capabilities. Increased oxidative stress in the PVN plays a vital role in regulating heart function and maintaining sympathetic nerve activity in spontaneously hypertensive rats [33]. H_2_S inhibits oxidative stress and inflammation caused by hypertension. Moreover, it improves endothelial function and reduces hypertension [34]. In our study, we found that exogenous H_2_S treatment increased the production of T-AOC, SOD, and GSH-Px and reduced the accumulation level of MDA to protect *Dahl S* rats.

We also evaluated the expression of NF-κB pathway-associated proteins, including p50, p65, and p-p65 using western-blotting methods. NF-κB is considered a key pro-inflammatory transcription factor that is involved in the expression of various genes, including cytokines [35]. Under normal conditions, NF-κB is sequestered in the cytoplasm via its inhibitory proteins, IκBs [36]. A series of kinases are activated via one or several signaling pathways once cells receive extracellular stimulation. This leads to the phosphorylation or degradation of IκBs, which allows NF-κB to be released from the compound. Then, NF-κB translocates from the cytoplasm into the nucleus and specifically binds to the κB site of associated genes to regulate the transcription of related genes, such as various genes of the cytokines. In this study, we showed that NaHS treatment can significantly inhibit the activation of NF-κB induced by the HSD in the PVN of *Dahl S* rats. This is consistent with the finding that H_2_S exerts anti-inflammatory effects by inhibiting NF-κB signaling in high glucose-induced inflammation [37]. Other studies have also confirmed that NF-κB activation promotes the expression of PICs in the central nervous system [19, 38]. Activation of NF-κB is highly dependent on the p50/p65 protein heterodimer, and the level of expression of these proteins (p50, p65, and p-p65) in the signaling pathway is often related to local or systemic inflammation [39]. Our exploration of H_2_S treatment in rats with a long-term HSD indicated that peripheral application of H_2_S significantly downregulates the expression of p50, p65, p-p65, and p-p65/p65. H_2_S may relieve the inflammatory response in brain tissue through the mediating the downregulation of the expression of NF-κB pathway-related proteins in the PVN. As neuromodulators of the central nervous system, PICs are involved in regulating neuronal activity [16]. NF-κB proteins in the cytoplasm translocate into the nucleus following activation to promote the transcription of PICs genes, such as *TNF-α, IL-6,* and *IL-1β*. Thus, it seems logical that H_2_S reduces protein expression of the NF-κB, which eventually results in the decreased expression of PICs in the PVN. The increased PICs in the PVN regulate sympathetic nerve activity in the *Dahl S* rats, which ultimately contributes to the development of hypertension [40, 41]. We postulate that the HSD-induced hypertension in *Dahl S* rats partially by enhancing the activity of the signaling pathway of ROS/NF-κB/PICs in the PVN. The exogenous H_2_S application then attenuated the activity of this pathway in the PVN neurons, reduced the hyperactivity of the cardiovascular activity-related neurons in the PVN and peripheral sympathetic activity, which ultimately delayed the increase in BP in the *Dahl S* rats on an HSD. However, these signaling pathways and the role of H_2_S need to be further investigated, specifically using inhibitors of H_2_S-generating enzymes, such as cystathionine β-synthase, in central and peripheral tissue.

Apoptosis, or programmed cell death, is a highly regulated mechanism of cell death that plays a critical role in the normal development and maintenance of tissue homeostasis in multicellular organisms [42]. Apoptosis is regulated by apoptosis family proteins, which include pro-apoptotic protein Bax and antiapoptotic proteins Bcl-2, cleaved-caspase3, and cleaved-PARP [43]. Our results indicated that H_2_S decreases the apoptotic index, Bax/Bcl-2 ratios, and protein expression levels of cleaved-caspase3 and cleaved-PARP in PVN tissues that had increased under an HSD. Treatment with exogenous H_2_S reversed the damage caused by the HSD and protected *Dahl S* rats by decreasing the cell apoptotic level in the PVN.

**Fig. 8:**
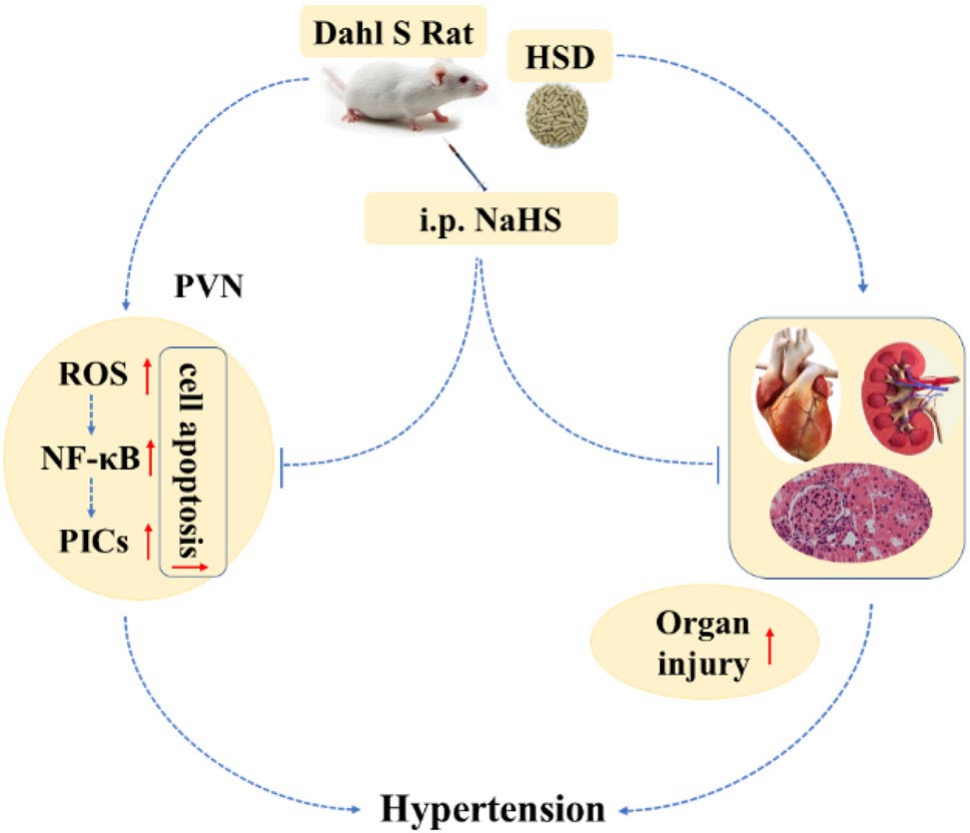
A proposed mechanism that exogenous hydrogen sulfide alleviates the hypertension induced by HSD in *Dahl S* rats. In one side, a long-term high salt diet can damage the peripheral organ such as heart and kidneys of the rats, in another side, it also can induce the oxidative stress, inflammatory response and cell apoptosis in PVN of the *Dahl S* rats. exogenous hydrogen sulfide released from the NaHS which was injected intraperitoneally can alleviate the hypertension induced by HSD through reducing the changes in the above indicators. *Dahl S Dahl S*alt sensitive, *HSD* high salt diet; *i.p.* intraperitoneal injection, *NaHS* sodium hydrosulfide, *ROS* reactive oxygen species, *PVN* paraventricular nucleus, *PICs* proinflammatory cytokines

This study confirmed the role of exogenous H_2_S administered by intraperitoneal injection in alleviating HSD-induced hypertension in *Dahl S* rats. We showed that H_2_S alleviates hypertension not only by relieving the injury of peripheral organs, such as kidneys and heart, caused by an HSD but also by reducing ROS/NF-κB/PIC signaling and cell apoptosis in the PVN (Fig. 8). Our findings provide a basis for initiating H_2_S-based therapy for hypertension associated with lifestyle-based eating habits, such as high salt intake. As a new gaseous neural mediator, H_2_S has been known to participate in a variety of physiological and pathophysiological processes in the body. The concentration of H_2_S or NaHS is a crucial factor that needs to be considered in this study. The relationship between biosafety and the dose of H_2_S application in the prevention or treatment of various diseases is needed for confirmation in future clinical research. With the accumulating evidence of H_2_S functions and its target effects in both animal and human studies, we anticipate that H_2_S will be valuable for the treatment in a variety of diseases because of its significant antioxidant, anti-inflammatory, anti-apoptotic, and neuroprotective properties.

## Data Availability

The datasets generated during and/or analyzed during the current study are available from the corresponding author on reasonable request.

## References

[1] Wu S, Wu B, Liu M, Chen Z, Wang W, Anderson CS, Sandercock P, Wang Y, Huang Y, Cui L, Pu C, Jia J, Zhang T, Liu X, Zhang S, Xie P, Fan D, Ji X, Wong KL, Wang L, China Stroke Study Collaborator (2019). Stroke in China: advances and challenges in epidemiology, prevention, and management. The Lancet Neurology.

[2] Wang YJ, Li ZX, Gu HQ, Zhai Y, Jiang Y, Zhao XQ, Wang YL, Yang X, Wang CJ, Meng X, Li H, Liu LP, Jing J, Wu J, Xu AD, Dong Q, Wang D, Zhao JZ, China Stroke Statistics Writing Colaborator (2020). China Stroke Statistics 2019: A report from the National Center for Healthcare Quality Management in Neurological Diseases, China National Clinical Research Center for Neurological Diseases, the Chinese Stroke Association, National Center for Chronic and Non-communicable Disease Control and Prevention, Chinese Center for Disease Control and Prevention and Institute for Global Neuroscience and Stroke Collaborations. Stroke and Vascular Neurology.

[3] Ye ZY, Li DP, Pan HL (2013). Regulation of hypothalamic presympathetic neurons and sympathetic outflow by Group II metabotropic glutamate receptors in spontaneously hypertensive rats. Hypertension.

[4] Qin C, Li J, Tang K (2018). The paraventricular nucleus of the hypothalamus: Development, function, and human diseases. Endocrinology.

[5] Beauchamp RO, Bus JS, Popp JA, Boreiko CJ, Andjelkovich DA (1984). A critical review of the literature on hydrogen sulfide toxicity. Critical Reviews in Toxicology.

[6] Whitcraft DD, Bailey TD, Hart GB (1985). Hydrogen sulfide poisoning treated with hyperbaric oxygen. Journal of Emergency Medicine.

[7] Powell CR, Dillon KM, Matson JB (2018). A review of hydrogen sulfide (H2S) donors: Chemistry and potential therapeutic applications. Biochemical Pharmacology.

[8] Kimura H (2014). The physiological role of hydrogen sulfide and beyond. Nitric Oxide.

[9] Olas B (2016). Medical functions of hydrogen sulfide. Advances in Clinical Chemistry.

[10] Kabil O, Banerjee R (2010). Redox biochemistry of hydrogen sulfide. Journal of Biological Chemistry.

[11] Duan XC, Guo R, Liu SY, Xiao L, Xue HM, Guo Q, Jin S, Wu YM (2015). Gene transfer of cystathionine beta-synthase into RVLM increases hydrogen sulfide-mediated suppression of sympathetic outflow via KATP channel in normotensive rats. The American Journal of Physiology-Heart and Circulatory Physiology.

[12] Peh MT, Anwar AB, Ng DS, Atan MS, Kumar SD, Moore PK (2014). Effect of feeding a high fat diet on hydrogen sulfide (H2S) metabolism in the mouse. Nitric Oxide.

[13] Wu D, Gao B, Li M, Yao L, Wang S, Chen M, Li H, Ma C, Ji A, Li Y (2016). Hydrogen sulfide mitigates kidney injury in high fat diet-induced obese mice. Oxidative Medicine and Cellular Longevity.

[14] Huang P, Shen Z, Liu J, Huang Y, Chen S, Yu W, Wang S, Ren Y, Li X, Tang C, Du J, Jin H (2016). Hydrogen sulfide inhibits high-salt diet-induced renal oxidative stress and kidney injury in Dahl rats. Oxidative Medicine and Cellular Longevity.

[15] Huang P, Shen Z, Yu W, Huang Y, Tang C, Du J, Jin H (2017). Hydrogen sulfide inhibits high-salt diet-induced myocardial oxidative stress and myocardial hypertrophy in Dahl rats. Frontiers in Pharmacology.

[16] Jiang E, Chapp AD, Fan Y, Larson RA, Hahka T, Huber MJ, Yan J, Chen QH, Shan Z (2018). Expression of proinflammatory cytokines is upregulated in the hypothalamic paraventricular nucleus of Dahl salt-sensitive hypertensive rats. Frontiers in Physiology.

[17] Liang YF, Zhang DD, Yu XJ, Gao HL, Liu KL, Qi J, Li HB, Yi QY, Chen WS, Cui W, Zhu GQ, Kang YM (2017). Hydrogen sulfide in paraventricular nucleus attenuates blood pressure by regulating oxidative stress and inflammatory cytokines in high salt-induced hypertension. Toxicology Letters.

[18] Larson RA, Gui L, Huber MJ, Chapp AD, Zhu J, LaGrange LP, Shan Z, Chen QH (2015). Sympathoexcitation in ANG II-salt hypertension involves reduced SK channel function in the hypothalamic paraventricular nucleus. The American Journal of Physiology-Heart and Circulatory Physiology.

[19] Qi J, Yu XJ, Fu LY, Liu KL, Gao TT, Tu JW, Kang KB, Shi XL, Li HB, Li Y, Kang YM (2019). Exercise training attenuates hypertension through TLR4/MyD88/NF-kappaB signaling in the hypothalamic paraventricular nucleus. Frontiers in Neuroscience.

[20] Cao X, Bian JS (2016). The role of hydrogen sulfide in renal system. Frontiers in Pharmacology.

[21] Zucker IH, Xiao L, Haack KK (2014). The central renin-angiotensin system and sympathetic nerve activity in chronic heart failure. Clinical Science (London).

[22] Han SJ, Noh MR, Jung JM, Ishii I, Yoo J, Kim JI, Park KM (2017). Hydrogen sulfide-producing cystathionine gamma-lyase is critical in the progression of kidney fibrosis. Free Radical Biology and Medicine.

[23] Wang Y, Xing QQ, Tu JK, Tang WB, Yuan XN, Xie YY, Wang W, Peng ZZ, Huang L, Xu H, Qin J, Xiao XC, Tao LJ, Yuan QJ (2019). Involvement of hydrogen sulfide in the progression of renal fibrosis. Chinese Medical Journa (England).

[24] Zhang DD, Liang YF, Qi J, Kang KB, Yu XJ, Gao HL, Liu KL, Chen YM, Shi XL, Xin GR, Fu LY, Kang YM, Cui W (2019). Carbon monoxide attenuates high salt-induced hypertension while reducing pro-inflammatory cytokines and oxidative stress in the paraventricular nucleus. Cardiovascular Toxicology.

[25] Aziz NM, Elbassuoni EA, Kamel MY, Ahmed SM (2020). Hydrogen sulfide renal protective effects: Possible link between hydrogen sulfide and endogenous carbon monoxide in a rat model of renal injury. Cell Stress Chaperones.

[26] Chen Y, Jin S, Teng X, Hu Z, Zhang Z, Qiu X, Tian D, Wu Y (2018). Hydrogen sulfide attenuates LPS-induced acute kidney injury by inhibiting inflammation and oxidative stress. Oxidative Medicine and Cellular Longevity.

[27] Choi EK, Park SH, Lim JA, Hong SW, Kwak KH, Park SS, Lim DG, Jung H (2018). Beneficial role of hydrogen sulfide in renal ischemia reperfusion injury in rats. Yonsei Medical Journal.

[28] Du Y, Liu XH, Zhu HC, Wang L, Wang ZS, Ning JZ, Xiao CC (2019). Hydrogen sulfide treatment protects against renal ischemia-reperfusion injury via induction of heat shock proteins in rats. Iranian Journal of Basic Medical Sciences.

[29] Whiteman M, Cheung NS, Zhu YZ, Chu SH, Siau JL, Wong BS, Armstrong JS, Moore PK (2005). Hydrogen sulphide: a novel inhibitor of hypochlorous acid-mediated oxidative damage in the brain?. Biochemical and Biophysical Research Communications.

[30] Abe K, Kimura H (1996). The possible role of hydrogen sulfide as an endogenous neuromodulator. Journal of Neuroscience.

[31] Kimura Y, Kimura H (2004). Hydrogen sulfide protects neurons from oxidative stress. FASEB Journal.

[32] Umemura K, Kimura H (2007). Hydrogen sulfide enhances reducing activity in neurons: Neurotrophic role of H2S in the brain?. Antioxidants & Redox Signaling.

[33] Yu XJ, Suo YP, Qi J, Yang Q, Li HH, Zhang DM, Yi QY, Zhang J, Zhu GQ, Zhu Z, Kang YM (2013). Interaction between AT1 receptor and NF-kappaB in hypothalamic paraventricular nucleus contributes to oxidative stress and sympathoexcitation by modulating neurotransmitters in heart failure. Cardiovascular Toxicology.

[34] Li J, Teng X, Jin S, Dong J, Guo Q, Tian D, Wu Y (2019). Hydrogen sulfide improves endothelial dysfunction by inhibiting the vicious cycle of NLRP3 inflammasome and oxidative stress in spontaneously hypertensive rats. Journal of Hypertension.

[35] Yin J, Duan JL, Cui ZJ, Ren WK, Li TJ, Yin YL (2015). Hydrogen peroxide-induced oxidative stress activates NF-kappa B and Nrf2/Keap1 signals and triggers autophagy in piglets. RSC Advances.

[36] Yin J, Ren WK, Wu XS, Yang G, Wang J, Li TJ, Ding JN, Cai LC, Su DD (2013). Oxidative stress-mediated signaling pathways: A review. Journal of Food, Agriculture and Environment.

[37] Zhou X, Feng Y, Zhan Z, Chen J (2014). Hydrogen sulfide alleviates diabetic nephropathy in a streptozotocin-induced diabetic rat model. Journal of Biological Chemistry.

[38] Qi J, Yu XJ, Shi XL, Gao HL, Yi QY, Tan H, Fan XY, Zhang Y, Song XA, Cui W, Liu JJ, Kang YM (2016). NF-kappaB blockade in hypothalamic paraventricular nucleus inhibits high-salt-induced hypertension through NLRP3 and caspase-1. Cardiovascular Toxicology.

[39] Wang C, Han J, Li DJ, Yang Z, Zhang L (2017). Protective effects of hydrogen sulfide on portal hypertensive vasculopathy in rabbits by activating AKT-NF-kappaB pathway. Journal of Huazhong University of Science and Technology Medical Sciences.

[40] Qi J, Zhao XF, Yu XJ, Yi QY, Shi XL, Tan H, Fan XY, Gao HL, Yue LY, Feng ZP, Kang YM (2016). Targeting interleukin-1 beta to suppress sympathoexcitation in hypothalamic paraventricular nucleus in Dahl salt-sensitive hypertensive rats. Cardiovascular Toxicology.

[41] Shi P, Raizada MK, Sumners C (2010). Brain cytokines as neuromodulators in cardiovascular control. Clinical and Experimental Pharmacology and Physiology.

[42] Sorenson CM (1998). Life, death and kidneys: Regulation of renal programmed cell death. Current Opinion in Nephrology and Hypertension.

[43] He Y, Fang X, Shi J, Li X, Xie M, Liu X (2020). Apigenin attenuates pulmonary hypertension by inducing mitochondria-dependent apoptosis of PASMCs via inhibiting the hypoxia inducible factor 1alpha-KV1.5 channel pathway. Chemico-Biological Interactions.

